# Deciphering Brain Function by Miniaturized Fluorescence Microscopy in Freely Behaving Animals

**DOI:** 10.3389/fnins.2020.00819

**Published:** 2020-08-11

**Authors:** Sarah Malvaut, Vlad-Stefan Constantinescu, Harold Dehez, Sead Doric, Armen Saghatelyan

**Affiliations:** ^1^CERVO Brain Research Center, Quebec City, QC, Canada; ^2^Department of Psychiatry and Neuroscience, Universite Laval, Quebec City, QC, Canada; ^3^Doric Lenses Inc., Quebec City, QC, Canada

**Keywords:** miniature endoscopes, mini-endoscopic imaging, animal behavior, GRIN lenses, GCaMP, GECI, Ca^2+^ imaging

## Abstract

Animal behavior is regulated by environmental stimuli and is shaped by the activity of neural networks, underscoring the importance of assessing the morpho-functional properties of different populations of cells in freely behaving animals. In recent years, a number of optical tools have been developed to monitor and modulate neuronal and glial activity at the protein, cellular, or network level and have opened up new avenues for studying brain function in freely behaving animals. Tools such as genetically encoded sensors and actuators are now commonly used for studying brain activity and function through their expression in different neuronal ensembles. In parallel, microscopy has also made major progress over the last decades. The advent of miniature microscopes (mini-microscopes also called mini-endoscopes) has become a method of choice for studying brain activity at the cellular and network levels in different brain regions of freely behaving mice. This technique also allows for longitudinal investigations while animals carrying the microscope on their head are performing behavioral tasks. In this review, we will discuss mini-endoscopic imaging and the advantages that these devices offer to research. We will also discuss current limitations of and potential future improvements in mini-endoscopic imaging.

## Introduction

Understanding how brain functions are shaped by experience and, in turn, how neural networks regulate animal behavior has always been of great interest to the scientific community as well as the general public. One of the long-term aims of neuroscience research is to understand how specific spatio-temporal activity patterns emerging from local neural networks regulate specific animal behaviors. Recording neural activity at the microcircuit level from distinct subsets of neurons and glia in freely behaving animals has thus become essential for studying how experience is represented and processed by the brain. The function of these networks can be interrogated using extracellular multi-electrode recordings (Nicolelis and Ribeiro, 2002), large-scale brain imaging techniques such as functional magnetic resonance imaging (fMRI), voltage-sensitive dye-based imaging, intrinsic optical signal imaging, and positron emission tomography (PET) (Grinvald et al., 1988; Shoham et al., 1999; Logothetis et al., 2002), or *in vivo* Ca^2+^ imaging using one- and two-photon mini-endoscopes (Dombeck et al., 2007; Kerr et al., 2007; Grewe and Helmchen, 2009). The technique of choice for these types of experiments has mostly involved taking electrophysiological recordings in either awake head-restrained or freely behaving rodents (Wilson and Mcnaughton, 1993; Houweling and Brecht, 2008; Harvey et al., 2009; Niell and Stryker, 2010). This approach has provided a great number of important insights into how neuronal ensembles orchestrate behavior such as the discovery of spatial representation by place and grid cells, for instance (O’keefe and Dostrovsky, 1971; Hafting et al., 2005). However, despite the tremendous value of this technique, the number of cells that can be recorded at a given time is limited by the small number of electrodes that can be used simultaneously. In theory, increasing the number of electrodes should make it possible to record electrophysiological activity in more cells, but the spacing between the electrodes is limited. Electrophysiological recordings also exclude electrically silent cells from the subsequent analysis of network dynamics (Shoham et al., 2006). To address some of these caveats, researchers can now take advantage of dense recordings using multi-tetrode arrays or silicone probes that make it possible to take recordings from large neuronal populations rather than from individual cells. Such large population-level recordings can be used to address questions that otherwise could not have been investigated using data from individual cell recordings (Csicsvari et al., 2003; Harris et al., 2003; Buzsaki, 2004; Dupret et al., 2010; Stensola et al., 2012). However, even with advances in electrophysiological recordings of large subsets of cells, there remain some important aspects that cannot be studied using such techniques. Although electrophysiology allows for distinct cell populations to be differentiated based on waveform shapes (Trainito et al., 2019), it is much more challenging to assess the individual contribution of each subset of recorded cells to the observed network dynamics. Recordings from subcellular structures such as dendrites can be also obtained in freely behaving mice using electrophysiological approaches (Moore et al., 2017). By taking advantage of the immune response that follows a tetrode implant, which allows a dendrite to get trapped between the tetrode tips and the glial encapsulation, it is possible to measure the dendritic membrane voltage in freely behaving rodents (Moore et al., 2017). This methodology could also provide important insights into the electrophysiological correlates of dendritic action potential integration, but has a low success rate and low yield.

The study of brain function has taken a step further with the advent of new optical tools designed to investigate neuronal activity at the protein, cellular, or network level. These include genetically encoded Ca^2+^ indicators (GECIs). Ca^2+^ is an important second messenger involved in the homeostasis of essentially all cells in the brain (Clapham, 1995; Berridge et al., 2000) and GECI-recorded Ca^2+^ fluctuations can be used to study the activity and coding of neural ensembles in basal conditions and in response to stimuli (Tian et al., 2012; Lin and Schnitzer, 2016). Many different versions of GECIs exist, and their repertoire and properties are constantly improving (Dana et al., 2016, 2019; Shen et al., 2020). The green and red Ca^2+^ indicators (GCaMP and RCaMP, respectively) are the most common (Lin and Schnitzer, 2016; Shen et al., 2020). More recently, a quadricolor GECI suite (XCaMP) (Inoue et al., 2019) and a near-infrared Ca^2+^ indicator (Qian et al., 2019) were designed to further expand the palette of GECIs and to study more complex neural dynamics. Using viral or transgenic labeling, GECIs can be expressed all over the brain or in specific neuronal/glial subtypes and are now widely used in the context of learning, memory formation, and plasticity (Jercog et al., 2016). They also allow for long-term investigations of brain activity for up to several weeks and even months (Jercog et al., 2016). In addition to GECIs, current efforts to develop genetically encoded voltage indicators (GEVIs) will eventually make it possible to monitor individual spikes during high-frequency trains of action potentials or subthreshold changes in the membrane potential in selected populations of cells (Bando et al., 2019). This will further increase the palette of functional assessments of neural networks during animal behavior. In addition to functional changes, several morphological modifications such as spine formation/elimination, the dynamics of glia processes, and microglia migration occur in response to environmental stimuli. Furthermore, several regions of the adult brain retain the capacity to renew their cellular populations during injury (Torper and Gotz, 2017) or under homeostatic conditions through adult neurogenesis (Gengatharan et al., 2016; Obernier and Alvarez-Buylla, 2019; Vicidomini et al., 2020). All these morphological changes can be also monitored *in vivo* using live imaging approaches (Berry and Nedivi, 2017; Pilz et al., 2018; Wallace et al., 2020).

*In vivo* imaging of morpho-functional changes in zebra fish larvae and nematodes has been performed in whole organisms (Ahrens et al., 2012; Nguyen et al., 2016). In rodents, *in vivo* imaging of a given brain region has traditionally been performed by two-photon microscopy in anesthetized or awake head-restrained animals performing specific behavioral tasks. While this allows for long-term and longitudinal recording of brain activity, it is limited to a restricted number of behavioral tasks and cannot be used to study deep brain structures unless the overlying brain regions are removed, which may affect cellular activity and animal behavior. The last two decades saw the advent of miniaturized fluorescent microscopes (also called mini-microscopes or mini-endoscopes) that enable *in vivo* recordings of brain structure and function of freely behaving animals to be taken at the cellular and network levels. These devices, which are similar to benchtop microscopes in their optical designs, contain miniaturized optical parts (mirrors, filters, or even a camera) and can be carried by animals that freely displace in an arena. Displays and recordings of acquired images are then computer assisted, allowing for the modulation of different parameters such as acquisition frame rate, exposure, and illumination power, without any additional manipulation of the recorded animal. Mini-endoscopes have evolved considerably in recent years since the first miniaturized versions were developed (Helmchen et al., 2001) and are still being refined and improved. They have helped provide answers to several unresolved questions regarding brain activity and structure. In this review, we will first present the various models of currently available mini-endoscopes and provide an overview of their specifications, advantages, and limitations. We will then discuss the *in vivo* applications of mini-endoscopy and the limitations of existing devices as well as future avenues for improving mini-endoscopic microscopy.

## Current State of the Art for Recording Brain Activity and Structure Using Mini-Endoscopes

### One-Color Mini-Endoscopes

The first versions of mini-endoscopes were based on two-photon excitation, and some microscope components such as lasers and photomultipliers (PMT) were converted to transmit the excitation light to the head of the animal or to collect emissions through optic fibers. These systems were used for imaging at the surface of the brain (Helmchen et al., 2001; Helmchen, 2002; Flusberg et al., 2005). They were bulky and heavy (25 g, 70 mm high), limiting applications involving naturalistic behavior. Also, the devices could not compensate very well for strong motion artifacts (Helmchen et al., 2001; Helmchen, 2002). Subsequent improvements led to the creation of lighter portable two-photon excitation microscopes (Gobel et al., 2004; Flusberg et al., 2008; Sawinski et al., 2009) that were used to image neural networks in freely moving mice and rats. A promising approach using a portable two-photon excitation-scanning microscope that weighs as little as 2 g was recently introduced to study activity in the entorhinal cortex of freely behaving mice (Zong et al., 2017; Rowland et al., 2018). These systems provide the obvious advantages of two-photon excitation microscopy such as a small excitation volume, increased tissue penetration, reduced phototoxicity, and the possibility of imaging subcellular structures. However, the low two-photon absorption cross-section also imposes high spatial (small focal spot) and temporal (short laser pulse) constraints on two-photon microscopy systems. To get sufficient excitation intensity at focus, it requires high numerical aperture diffraction-limited optics as well as expensive table-top femtosecond pulse lasers with proper dispersion compensation. Laser scanning two-photon microscopes should also be properly stabilized to avoid motion artifacts as the pixels of each image are not recorded simultaneously. It is thus more challenging to use two-photon excitation in a context of mini-endoscopes, suggesting that miniaturized epifluorescence microscopy tends to represent a simpler and more affordable method for imaging activity in neural networks at the cellular level, particularly in deep brain regions while animals are freely behaving.

Several groups have taken advantage of small optical rod lenses [gradient refractive index (GRIN) lenses] (Jung and Schnitzer, 2003; Levene et al., 2004) to build mini-endoscopes. These lenses have a short working distance and can be fixed either at the surface of the brain or inserted into deep brain regions to perform imaging in the superficial or deep brain regions, respectively (Helmchen et al., 2001; Helmchen, 2002; Flusberg et al., 2005). GRIN lenses, which can have different diameters and customizable lengths, have been used for *in vivo* imaging of previously unreachable deep brain structures such as the substantia nigra, the hypothalamus, the parabrachial nucleus, and the basal forebrain (Bocarsly et al., 2015; Fu et al., 2019; Patel et al., 2019). GRIN lenses are usually flat at the tip, making it possible to image cells just below the tip. However, they can also be coupled with a prism mirror allowing for “side-view” imaging. This configuration, in our experience, significantly reduces tissue damage and immune responses compared to blunt probes. In line with this, less tissue damage and better neuronal preservation is observed for well-sharpened and small tip angle silicone probes used for *in vivo* electrophysiological recordings (Edell et al., 1992). The GRIN coupled with a prism has been used for imaging neurons located in different cortical layers (Gulati et al., 2017).

Gradient refractive index implantation is minimally invasive, and the presence of the lens in an animal’s brain does not alter the locomotion or behavior of the animal in selected tasks commonly used in neuroscience research (Lee et al., 2016), although this largely depends on the diameter and type of GRIN lens (Resendez et al., 2016). Larger diameter (1 mm) GRIN lenses have been described as being more suitable for an implantation in superficial brain regions such as the cortex or the hippocampus, whereas smaller diameter GRINs are more suitable for deeper brain regions such as the hypothalamus or the ventral tegmental area (Resendez et al., 2016). Slow insertion of probes during implantation or using probes coupled with a prism can minimize possible compression of the brain, restricting it to the front of the GRIN and reducing possible damage (Edell et al., 1992; Levene et al., 2004). For deep brain region imaging, a track in the brain is often created using a sharp needle or blade. Brain tissue aspiration is also used in some cases (Resendez et al., 2016; Gulati et al., 2017; Gonzalez et al., 2019; De Groot et al., 2020). The inflammatory response resulting from implant insertion normally largely clears up after 4 weeks (Bocarsly et al., 2015), or less for sharp-ended implants (Edell et al., 1992; Turner et al., 1999). This observation highlights the need to start recording with miniature endoscopes several weeks post-implantation. A minimal period ranging from 2 to 4 weeks is required before acquiring data using GRIN lenses (Barbera et al., 2019; Fu et al., 2019; Gonzalez et al., 2019). To confirm the correct implantation of a GRIN lens, a *post hoc* analysis in post-mortem brain tissues can be used. This step makes it possible to adapt the stereotaxic coordinates used in different animals (Lee et al., 2016; Resendez et al., 2016). When viral vectors are injected in a defined brain region a few weeks before GRIN implantation, changes in background fluorescence may be also used to ascertain the correct positioning.

As for animals becoming habituated to the imaging device, it is common practice to try to reduce the weight of mini-endoscopes as much possible so as to not interfere with naturalistic behavior of the animals (Resendez et al., 2016). Chronic GRIN implants do not alter animal performance in standard behavioral procedures such as the rotarod test or the Morris water maze (Lee et al., 2016). However, no comparative studies have been reported with regard to the adaptation of animals to mini-endoscopes of different weights and configurations. Further behavioral investigations will be required to accurately evaluate the maximal weight that small rodents like mice can carry on their head without an impact on their behavior and to compare different mini-endoscopes, some of which are bulkier than others. Also, not all available versions of mini-endoscopes can reach very lateral or anterior brain regions such as the olfactory bulb. In fact, due to size constraints, placing a mini-endoscope in such regions could potentially alter the normal behavior of the animals or even their vision if placed too close to their eyes. Smaller footprint mini-endoscopes may help to resolve this issue.

An important step in the conception of mini-endoscopes is thus the miniaturization of microscope parts. Ghosh et al. (2011), in their first version of a fully miniaturized epifluorescence mini-endoscope, replaced larger external light sources (e.g., Xenon lamps, mercury lamps, high power LEDs, and lasers) with smaller internal LEDs that are efficient and low-cost alternatives. This approach is now widely used in different open source mini-endoscopes designed to image in freely behaving rodents or songbirds. These include the UCLA Miniscope (Cai et al., 2016), the National Institute of Drug Abuse MiniScope (Barbera et al., 2016), the Boston University FinchScope that was developed for imaging in songbirds (Liberti et al., 2017), and the University of Toronto CHEndoscope (Jacob et al., 2018). Although very practical in terms of cost, availability, and size, installing LEDs directly in the microscope body can have some drawbacks. Because of its location in the microscope body, maximum output power is limited in order to minimize potential excessive heat on the animal’s head at higher powers normally used for optogenetic stimulations. To alleviate these issues, some commercial one-color mini-endoscopes can be connected to adjustable, exchangeable external light sources such as lasers, laser pumped incoherent sources (Ce:YAG), or LEDs using an optical fiber. However, this has the disadvantage of adding an extra optical fiber above the animal’s head.

Mini-endoscopes also take advantage, in their design, of off-the-shelf miniature electronic components such as complementary metal oxide semiconductor (CMOS) sensors for image detection. These sensors support high acquisition frame rates (higher than 30 Hz) and can, in some cases, be coupled to a microphone to record songbird vocalizations (Liberti et al., 2017). Improved versions of mini-endoscopes are currently available from commercial sources (Doric Lenses, Inscopix, and Neurescence) as well as open-source platforms (UCLA Miniscope, NINscope, and FinchScope).

It should be also mentioned that because of their miniaturization and their use in freely behaving animals, device failure may also occur. Unlike tabletop microscopy systems, miniaturized fluorescence microscopes are operated in challenging environments, with the animal moving in different directions and potentially applying some constraints on the microscope and optical fibers. Several approaches have been investigated to reduce the risks of experiment interruption due to microscope failure, including sturdier bodies in hard plastic (Cai et al., 2016) and metal frames (Zong et al., 2017), protected electronics, and connectorized systems with interchangeable cables (Barbera et al., 2016; Cai et al., 2016; Liberti et al., 2017; Jacob et al., 2018; De Groot et al., 2020). Optical components used in miniature endoscopes can be costly and, because of their size, failure can happen during surgical implantation or extraction. To counteract this, Bocarsly et al. (2015) proposed a version of the device, including the implantation in animal’s brain of a guide cannula, allowing for the insertion of a GRIN lens for each imaging session. However, it should be noted that the resulting diameter of the imaging probe is larger, which has an impact on brain tissue, as previously reported (Bocarsly et al., 2015; Resendez et al., 2016).

### Two-Color Mini-Endoscopes

Functional and structural imaging in a given brain region was, for a long time, restricted to a general cell population or a subset of cells expressing a particular Ca^2+^ indicator or fluorescent marker. More recently, two-color versions of mini-endoscopes have been developed that allow two different fluorophores with different emission and excitation wavelengths to be imaged. However, GRIN lenses have large chromatic aberrations that induce a shift (sometimes up to several tens of microns) in the imaged focal plane when comparing the fluorescence of two different fluorophores. To counteract these aberrations, one solution is to place two CMOS sensors in the microscope body at a specific location to compensate for the focal shift induced by the GRIN lens when imaging the two spectrally distinct fluorophores. Another solution for counteracting chromatic aberrations is to use achromatic lenses. The availability of several versions and colors of GECIs (Dana et al., 2016, 2019; Shen et al., 2020), each with its own emission/excitation spectra, means that it is now possible to image using both green and red Ca^2+^ indicators with similar kinetics (Shen et al., 2020). Two-color mini-endoscopes have a number of advantages. First, when combined with Ca^2+^ indicators of spectrally distinguishable colors (GCaMP and RCaMP, for instance), it is possible to compare the activity of two different cell populations in the same brain region of freely behaving animals. In addition, two-color mini-endoscopes can be used for motion correction. In fact, *xy* and *z* drift can occur when animals are moving, which may create some bias in data analysis and interpretation. This issue can be addressed by performing functional Ca^2+^ imaging combined with morphological fluorescent marker imaging followed by open-source plugin or software processing for motion and drift correction. These systems can be also used for imaging cell populations that can be defined only based on the combinatory genetic labeling approaches, leading to the expression of two different fluorophores (or sensors) by the same cell. Two-color mini-endoscopes can be also used for assessing the interplay between different cellular populations in a given brain region. It should be noted, however, that because of their optical design, two-color mini-endoscopes are bulkier and a longer habituation period for the animal may be required before starting the experiments.

### Combined Optogenetic and Imaging Mini-Endoscopes for Studying Brain Connectivity and Disease Pathogenesis

The discovery that neuronal activity can be modulated using light-sensitive ion channels or opsins was a breakthrough in the field of neuroscience (Kim et al., 2017). *In vivo* optogenetic approaches are now widely used to modulate neuronal activity and to investigate the impact on animal behavior (Kim et al., 2017). It is possible to modulate neuronal activity by either stimulating or inhibiting the activity of a cell population in a given structure while simultaneously imaging the responses of the same or neighboring populations of cells in the same structure or even in a very different, remote structure (Stamatakis et al., 2018). However, the opsin and Ca^2+^ indicators have to be carefully chosen as crosstalk between the channels may affect the recordings (Stamatakis et al., 2018). In addition, the imaging light source attenuates the optogenetic response (Stamatakis et al., 2018), which means that care must be taken in choosing the excitation light source for imaging in order to provide a better signal-to-noise ratio and minimal crosstalk.

De Groot et al. (2020) developed the NINscope, which can be used to simultaneously record and optogenetically stimulate two distant brain structures. The authors reduced the footprint of the mini-endoscope, making it possible to record the Ca^2+^ activity of a subset of cells in a given brain region while optogenetically stimulating cells in many other brain areas using small LEDs installed on the surface of the brain (De Groot et al., 2020). For experiments requiring optogenetic manipulation of deeper brain regions that cannot be targeted with the NINscope, a micro-LED can be implanted inside the brain. The stimulation can then be synchronized with the endoscope imaging (Shin et al., 2017). Combined optogenetic and imaging mini-endoscope baseplates composed of a GRIN relay lens for fluorescence imaging and an optical fiber for opsin activation are also commercially available. These systems offer a customizable length and distance between the imaging cannula and the implanted optical fiber. We used one of these mini-endoscope baseplates to optogenetically induce α-synuclein aggregation in the substantia nigra and to monitor changes in striatal neurons using Ca^2+^ imaging (Bérard et al., 2019).

### Multi-Region Imaging With Mini-Endoscopes

The NINscope developed by De Groot et al. (2020) also allows the simultaneous and synchronous imaging of two separate brain regions because of its reduced footprint. This is of particular relevance in a context of brain connectivity as the activity of two distinct regions can be correlated at the cellular level depending on the selected behaviors or stimuli. Simultaneous recording using two separate mini-endoscopes can also be performed in both hemispheres of the same structure (Gonzalez et al., 2019; De Groot et al., 2020). These recordings can be used to investigate the lateralization of brain activity (Gonzalez et al., 2019) and to compare, in the same subject, the activity of two hemispheres to which a different treatment may have been applied. A device allowing for simultaneous imaging of different brain regions is now also commercially available. In fact, because of it small footprint, the fiber bundle-based device from Neurescence (quartet mini-microscope) can record neuronal activity from up to four different brain regions as well as from different animals that are socially interacting, for instance.

### Mini-Endoscopes With Dual-Modality Recordings

Mini-endoscopes also allow for dual-modality recording of neuronal activity. Yashiro et al. (2017) used a mini-endoscope system coupled with electrophysiological recordings of neural activity to make simultaneous optical recordings from large neuronal subsets. Such simultaneous optical and electrical recordings may prove to be extremely useful in correlating electrically recorded phenomena (multi-unit activity and local field potentials) with optically recorded Ca^2+^ fluctuations. Combined electrical and Ca^2+^ signal recordings from a single fluorescent neuron *in vivo* have also been made and allow the spiking activity of recorded cells to be correlated with changes in Ca^2+^ dynamics (Lechasseur et al., 2011). In addition to combining imaging and electrophysiological recordings, commercially available dual implant baseplates composed of a GRIN lens and an optical fiber allow Ca^2+^ imaging and photometry recordings in two different brain regions located as close together as 1 mm to be performed. Another potential new application for mini-endoscopes involves combining Ca^2+^ imaging with intrinsic optical signals (Senarathna et al., 2019). This device can be used to monitor neuronal activity in large areas of the brain and subtract the hemodynamic signal, providing insights into neurovascular coupling (Senarathna et al., 2019). Other types of dual-modality recording mini-endoscopes are also available, such as those that allow for combined Ca^2+^ imaging and bird vocalization recording (Liberti et al., 2017). It is likely that in coming years other dual-modality mini-endoscopes will be developed. These systems could allow for combining imaging and local drug delivery via integrated cannula, Ca^2+^ activity recordings and respiration/sniffing assessments, or simultaneous Ca^2+^ and voltage imaging, for instance. This in turn will make it possible to perform multi-modal assesments of neural networks during unrestricted animal behavior.

### Electronic Modulation of the Field of View: eFocus Mini-Endoscopes

A non-negligible drawback of traditional mini-endoscopes was, for a long time, the inability to change the focus prior to or during image acquisition, limiting imaging to a single focal plane (Barretto and Schnitzer, 2012; Hamel et al., 2015). Indeed over time, following consecutive imaging sessions, the focal plane can change slightly and tracking the same cells can become challenging. To counteract this, the first versions of commercial and open-source mini-endoscopes offered the possibility to manually and mechanically adjust the image focus with a slider, screw, or turret before starting a recording, which resulted in additional manipulations of both the device and the animal (Ghosh et al., 2011; Barbera et al., 2016; Cai et al., 2016; Liberti et al., 2017; Jacob et al., 2018). To deal with this issue, updated electronically focused versions of endoscopes have been released by several companies such as Inscopix and Doric Lenses or were built from open-source designs (e.g., UCLA miniscope version 4). These versions make it possible to adjust the focus remotely and thus avoid mechanical manipulations of the endoscope while the animals are freely behaving. To that end, several groups have taken advantage of different strategies. For example, electro-wetting liquid lenses, which are small devices containing two non-miscible liquids, have been used. They allow for the rapid deformation of the lenses and thus the electronic focusing of the field of view by applying a voltage (Grewe et al., 2011; Zou et al., 2015; Ozbay et al., 2018). Electronically tunable liquid crystal lenses have also been used (Bagramyan et al., 2017). These lenses, which are traditionally used in liquid crystal displays, consume little energy and are lighter than electro-wetting liquid lenses, two important parameters for improving mini-endoscopes without compromising their size and weight.

## *In Vivo* Applications of Mini-Endoscopic Imaging

As discussed above, two main approaches for optical brain imaging studies on live animals have emerged—those that require the experimental subjects to be head-fixed, such as two-photon excitation microscopy, and those that allow for unrestrained behavior, such as mini-endoscopes. Each technique has its share of advantages and drawbacks. Depending on the nature of the study, researchers can now use a variety of methods that can accommodate either imaging approach. New advancements in fluorescent reporter proteins, in particular GECIs and GEVIs, mean that neuroscientists can perform two-color fluorescence imaging and can distinguish the activity of a dual-labeled subset within a broad population of indicator-labeled cells (Chen et al., 2013; Inoue et al., 2015; Malvaut et al., 2017). The head-fixed imaging approaches that use high-performance lenses and the optical sectioning provided by two-photon microscopy allow for the interrogation of activity patterns during animal behavior at subcellular levels such as in dendrites, dendritic spines, and even axonal boutons (Petreanu et al., 2012; Xu et al., 2012; Boyd et al., 2015; Sheffield and Dombeck, 2015). The head-fixed paradigm can also make intracranial drug delivery easier (Nimmerjahn et al., 2009; Lovett-Barron et al., 2014). Behavioral assays, including adapted fear conditioning paradigms (Lovett-Barron et al., 2014), controlled delivery of sensory stimuli (Verhagen et al., 2007; Carey et al., 2009; Andermann et al., 2011; Blauvelt et al., 2013; Patterson et al., 2013; Miller et al., 2014), and perceptual discrimination tasks (Andermann et al., 2010; Komiyama et al., 2010; O’connor et al., 2010) can now be done in a head-restrained configuration to study changes at subcellular levels during complex behavioral tasks. Another exciting avenue for head-restrained optical imaging in live animals involves the use of virtual reality approaches (Harvey et al., 2009, 2012; Dombeck et al., 2010), which could provide new information about an animal’s spatial navigation.

However, even with the enrichment of the behavioral repertoire that can be tested during head-restrained experiments, the array of behavioral manipulations is still restricted and is associated with the stress that the animals may encounter during head fixation and imaging. This stress can have an adverse effect on the outcome of the behavioral analysis and is likely to affect the neuronal activity patterns recorded (Thurley and Ayaz, 2017). Miniaturized head-mounted imaging devices were designed to circumvent these shortcomings. The main motivation for these devices was first and foremost their ability to record activity from many neurons in an animal that is unrestrained and that can display natural innate behavior as well as a broad array of social behaviors such as fighting, mating, care-giving, and other forms of interaction. The need to accurately monitor cell activity in freely behaving experimental subjects has led to recent advances in mini-endoscopic imaging that make it possible to record neuronal activity simultaneously from different brain regions (De Groot et al., 2020), to combine Ca^2+^ imaging and recordings of other modalities such as electrical recordings of neural activity (Yashiro et al., 2017) or sound vocalizations (Liberti et al., 2017), and to image activity in neural networks at different focal planes and in different neuronal populations using efocus and two-color versions of mini-endoscopes. Also, the versatility of mini-endoscopes enables cell activity to be tracked across a large array of behavioral paradigms established over the last decades (Morris, 1984; Graeff et al., 1998; Nadler et al., 2004).

The main *in vivo* application for mini-endoscopes is the imaging of GECIs in large subsets of neurons in freely behaving animals. As mini-endoscopes have many useful characteristics such as relative ease of use, scalability, and commercial availability, they have seen a significant spread to numerous research fields. The use of mini-endoscopes in neuroscience-related fields such as learning and memory, neuropsychiatric disorders, and drug discovery has enabled scientists to accurately describe temporal changes that neuronal circuits exhibit during memory formation and retention as well as modifications due to a pathology. The field of learning and memory has in particular been directly influenced by the development of mini-endoscopes. Tracking the formation and subsequent modification of cellular ensembles that retain learned information, or engrams, over long periods of time has allowed neuroscientists to answer questions about the coding dynamics underlying the neurobiology of long-term memory (Tian et al., 2009; Cai et al., 2016). Established behavioral assays coupled with long-term monitoring of cellular activity has revealed that activity-induced modifications in neuronal CREB (cAMP response element-binding protein) levels are encoded by a shared neural ensemble in the CA1 part of the hippocampus that links distinct contextual memories occurring close in time (Cai et al., 2016). Neuronal activity in the spinal cord of freely behaving mice has also been recorded using a vertebra-affixed mini-endoscope (Sekiguchi et al., 2016). Implanting a mini-endoscope in the spinal cord is technically more challenging than anchoring an implant on top of the skull due to the pronounced movement of the spine within the vertebral column. Although fewer cells can be imaged due the curvature of the spinal cord, these experiments showed that distinct cutaneous stimuli activate overlapping ensembles of dorsal horn neurons and that stimulus type and intensity are encoded at the single-cell level (Sekiguchi et al., 2016).

Mini-endoscopic applications are not limited to the field of learning and memory. Mouse models of diseases are very important for studying the neurobiological basis of a myriad of brain disorders. As most of these diseases are, to some extent, characterized by a deficit in information processing, imaging neural activity in transgenic and knockout mice could be very advantageous for elucidating the underpinnings of the disease mechanisms (Nestler and Hyman, 2010). For example, much more information about alterations in coding dynamics in animal disease models can be inferred from large-scale imaging in freely behaving animals than is possible with electrophysiological measurements. These approaches could also narrow the gap between molecular and behavioral analyses. This concept becomes clear in the context of Alzheimer’s disease (AD), where much progress in understanding the molecular pathology underlying the neurodegeneration observed in human patients has led to the emergence of different mouse models that exhibit one or more AD hallmarks (b-amyloid, presenilins, apolipoprotein E, and Tau) (Knowles et al., 2009; Sterniczuk et al., 2010; Filali et al., 2012; Youmans et al., 2012). However, little is known about how these molecular pathways influence population dynamics during memory encoding and retrieval. With the advent of mini-endoscopy, longitudinal imaging in different AD models during the performance of memory tests is now feasible and could shed new light on memory coding dysfunctions in AD as well as in other neurological and psychiatric disorders (Ziv and Ghosh, 2015). As in the case of AD, the use of mini-endoscopes in animal models of other neurodegenerative disorders and brain trauma may allow the early hallmarks of disease manifestation to be deciphered. These mini-endoscopes combined with optogenetic stimulation can also be used to induce molecular alterations in cells and study disease progression. For example, it is now possible to optogenetically induce the aggregation of α-synuclein in dopaminergic neurons in the substantia nigra and to study alterations in the nigrostriatal pathway and in the network activity of striatal neurons using longitudinal mini-endoscopic Ca^2+^ imaging (Bérard et al., 2019).

Drug discovery could also benefit from mini-endoscopic imaging. Although much research has focused on the pharmacological and molecular mechanisms of drugs at the cellular level, much less attention has been directed at understanding the effect of drugs on neuronal coding. Berdyyeva et al. (2014) showed that a GABA-A agonist used for treating insomnia reduces hippocampal neuronal firing in freely behaving mice. This result, although not surprising, shed light on the need to correlate neuronal dynamics with the effects of chronic drug administration and to identify the effects of the drugs on the overall neuronal network. Addressing these issues would further advance our ability to design future therapies for treating brain diseases (Ziv and Ghosh, 2015).

The capability of mini-endoscopes to record neural activity in freely behaving animals can be further enhanced by using optogenetic stimulations (Stamatakis et al., 2018) or by combining two different recording techniques such as extracellular electrophysiological recordings with Ca^2+^ imaging in freely behaving animals (Yashiro et al., 2017). Stamatakis et al. (2018) used a mini-endoscope that jointly records optical neural activity and light-induced alterations of cellular ensemble activity in the same field-of-view. Other researchers have used a device to optogenetically stimulate brain regions away from the imaging field-of-view (Shin et al., 2017; Bérard et al., 2019; Senarathna et al., 2019). Engineering advances in the manufacture of mini-endoscopes have led to a reduction in the weight and size of these devices, making it possible to simultaneously record cellular activities in different brain regions as was done using the NINscope (De Groot et al., 2020). Such types of experiments will yield new data subsets that can be used to analyze the extent to which lateralization occurs during behavior and learning and to track the network stability of multi-region cellular ensembles over long periods of time (Gonzalez et al., 2019).

Mini-endoscopes are mostly used to investigate *in vivo* brain function in freely behaving animals by Ca^2+^ imaging of cell populations in a given region and are evolving in parallel with improvements in GECIs. The use of such powerful tools will likely expand to the study of other processes underlying brain activity. For instance, an *in vivo* two-photon investigation of adult neurogenesis and, more particularly, stem cell division has been already performed in anesthetized head-restrained animals (Pilz et al., 2018). Mini-endoscopes could help unravel this process in freely behaving animals and to correlate stem cell division with naturalistic patterns of animal behavior. These experiments can be performed under homeostatic conditions in known neurogenic regions of the brain like the dentate gyrus or the subventricular zone, as well as after injury and neuronal regeneration. In line with this, mini-endoscopic imaging has shown that coupling pre-existing neurons to the cerebrovascular system regulates adult hippocampal neurogenesis (Shen et al., 2019) and that newborn neurons in the adult dentate gyrus migrate laterally first to increase their dispersion (Wang et al., 2019). The migration of neuronal progenitors and microglial cells following different neurodegenerative disorders and brain trauma can also be recorded by mini-endoscopic imaging. In addition, the use of miniaturized two-photon microscopes makes it possible to image Ca^2+^ activity in dendrites (Zong et al., 2017) and may be used in the future to image other sub-cellular structures. Future studies will certainly deepen our understanding of the cellular and molecular mechanisms operating in homeostatic and diseased conditions.

## Limitations and Future Avenues for Mini-Endoscopic Imaging

Given the experimentally proven benefits and versatility of mini-endoscopes for neuroscience research, it has become increasingly clear that the impact of these devices will grow exponentially. However, they still have some limitations and drawbacks and further improvements are needed. The weight and size of mini-endoscopes are crucial and should be further improved to enable unrestrained one- and two-photon mini-endoscopic imaging in freely behaving mice and other small animal models. Similarly, the footprint of mini-endoscopes should be reduced for multi-region imaging in order to record activity from adjacent brain areas. Another caveat is that most of the mini-endoscopes used in freely behaving animals need to be tethered to optical components that are too big and too heavy to be mounted on an animal’s head, which means that more complex social behaviors such as social interactions, mating, and mate aggression cannot be fully investigated due to the obvious physical constraints. The poor optical sectioning of one-photon mini-endoscopes and the major aberrations caused by their optical components (GRIN lenses) should also be taken into consideration and be improved. Below we discuss some of the avenues for addressing these issues.

### Optical Sectioning

Despite the advantages of wide-field epifluorescence mini-endoscopy (large field of view, small dimensions, and technical simplicity), these systems do not provide the optical sectioning of widely used tabletop systems such as confocal or two-photon microscopes or two-photon mini-endoscopes. As a consequence, image contrast in the focal plane is strongly attenuated by the out-of-focus fluorescent background when imaging densely labeled structures, especially in scattering media. The strong background limits neuronal and glial activity recording to the soma, as smaller structures such as dendrites, dendritic spines, and glial processes are too dim to be resolved. One of the key avenues for future developments is thus the integration and miniaturization of non-scanning optical sectioning systems already offered in large-scale microscopes.

Among non-scanning techniques, the tabletop version of the HiLo microscope has generated a great deal of interest (Lim et al., 2011; Lauterbach et al., 2015). The HiLo microscopy technique is based on epifluorescence microscopy and makes it possible to reject the out-of-focus fluorescent background by processing two images, one recorded with a uniform illumination and the other recorded with a high contrast pattern. HiLo microscopy provides high contrast images with optical sectioning similar to confocal microscopy at the expense of half the camera frame rate. The use of speckle illumination for the high contrast illumination has been proposed, as fast switching from uniform to speckle illumination can be obtained with the simple addition of a diffuser and a galvanometric scanner. HiLo microscopy has already been used for endoscopic applications using a flexible fiber bundle to deliver shaped illumination to the sample and to collect the fluorescence (Ford et al., 2012). Although fiber bundles allow images and illumination patterns to be relayed over long distances (up to meters), they are more rigid than the smaller diameter multi-mode fibers used to deliver light in experiments on freely behaving animals. They also have a small core fill factor, which results in low optical transmission. These properties limit their use for dimmer fluorescent signal recording in freely behaving small animals. Generating both illumination types in the microscope body at the expense of the size of the system is a good alternative to fiber bundles, and the technical simplicity of the HiLo microscopy technique makes it a good candidate for miniaturization.

More recently, it has been proposed that the light field microscopy (LFM) and seeded iterative demixing (SID) techniques can be integrated into miniaturized microscopes (Nobauer et al., 2017; Skocek et al., 2018). These computational methods only require minor hardware modifications to the microscope body by inserting a microlens array in the detection pathway of a miniaturized epifluorescence microscope. A 700 μm × 600 μm × 360 μm volume has been imaged at a frequency of 16 Hz with a discrimination threshold between cells of 15 microns. Despite the complex analytics and lower spatial resolution of this method, the reduced number of optical components and the extraction of a large volumetric amount of data makes it a good candidate for Ca^2+^ activity recording in freely behaving animals with improved optical sectioning.

As mentioned above, the first versions of mini-endoscopes were based on a two-photon excitation principle that provides superior optical sectioning (Helmchen et al., 2001; Helmchen, 2002; Flusberg et al., 2005). However, these systems are bulkier and heavier, which limits the behavioral paradigms that can be studied. In these versions, the laser scanning mechanism is either located proximally on the animal for higher resolution and sensitivity, or remotely and is connected to the animal with a fiber bundle to limit the number of optical components on the animal (Ozbay et al., 2015, 2018). To reduce the weight of the imaging systems, miniaturized scanning systems were developed based on micro-mechanical systems (MEMS), scanning mirrors (Piyawattanametha et al., 2006; Zong et al., 2017), and piezoelectric actuators (Engelbrecht et al., 2008). High resolution miniaturized two-photon mini-endoscopes capable of imaging a 130 μm × 130 μm-field of view at 40 Hz have been used to record Ca^2+^ activity in dendrites and spines in freely behaving animals (Zong et al., 2017). In this system, a hollow core photonic crystal fiber and an achromatic objective lens are used to minimize light pulse broadening and maintain the high intensity at focus essential for two-photon excitation. However, this work was done in the first layers of the brain (visual cortex). Minimizing pulse broadening remains one of the main challenges of deep brain imaging. To achieve this, the chromatic aberration of the GRIN lenses used for deep brain imaging has to be properly compensated without increasing the outer diameter of the implant. This will require the development of new minimally invasive achromatic optical probes. Another interesting approach to increase the imaging depth without using GRIN lenses is miniaturized three-photon microscopy. Klioutchnikov et al. (2020) recently designed a three-photon microscope capable of recording calcium transients at multiple cortical depths of up to 1120 microns in freely behaving animals. However, the weight of the device developed by Klioutchnikov et al. (2020) (around 5 g) restricts its use for now to bigger rodents such as rats.

Miniaturized confocal microscopes with a scanning system placed on the animal would provide optical sectioning without a complex laser system and non-linear effect management. The main drawback of confocal microscopy compared to two-photon microscopy is its lower excitation wavelength, which creates more scattering and higher phototoxicity. The chromatic aberrations of the optical system, including the GRIN relay lens, will also have to be properly compensated to ensure that the pinhole and object plane remain in conjugated planes.

### Optical Aberration Compensation

The high numerical aperture and the small outer diameter of GRIN lenses (0.5–1 mm) allow the imaging of structures located several millimeters deep in tissue with minimal invasiveness. In comparison, relay lenses made of optically homogenous materials with a similar numerical aperture are at least five times larger in diameter. Given this, GRIN lenses have become the preferred option for *in vivo* deep structure imaging. However, GRIN lenses also suffer from high intrinsic on-axis and off-axis optical aberrations (astigmatism and chromatic) and have the highest optical aberrations in miniaturized microscopy systems, leading to lower spatial resolutions and smaller effective fields of view, especially with high numerical aperture GRIN lenses. To solve this issue, adaptive optics methods have been used in two-photon mini-endoscopes. One of these methods allows the recovery of diffraction-limited resolution in a large field of view by measuring and correcting off-axis aberrations using pupil segmentation-based adaptive optics (Wang and Ji, 2012). Future developments in miniature microscopy would also benefit from the use of miniature tunable liquid crystal lenses and miniaturization of the phase modulators used in adaptive optics methods such as phase liquid crystal spatial light modulators. Adding adaptive optics to miniaturized microscopes would also help reduce the impact of light scattering in tissue. The inhomogeneity created by the different components of the sample (water, proteins, and lipids, etc.) distorts the fluorescence wavefront, resulting in lower resolution and contrast.

### Wireless Imaging Systems

With the development of tethered miniaturized microscopes, it became possible to correlate brain activity and behavior recordings. However, despite several attempts to reduce the impact of the cables on animal behavior (very flexible cables, motor-assisted rotary-joints, etc.), some complex behavioral studies such as social interaction studies with multiple animals cannot be performed with these systems due to cable entanglement. To overcome these issues, several wireless models have been proposed (Liberti et al., 2017; Barbera et al., 2019; Shuman et al., 2020). One of the main challenges in developing wireless systems is minimizing their size and weight by, for instance, reducing the size of the lithium polymer (LiPo) batteries. Several avenues have been explored to reduce power consumption and use lighter batteries: limiting the number of on-board electronic components with on-site recording (Barbera et al., 2019; Shuman et al., 2020) and using low resolution sensors to restrict the amount of transferred data (Liberti et al., 2017). Using a battery backpack for a better distribution of the weight has also been explored (Barbera et al., 2019). For example, Liberti et al. (2017) introduced the FinchScope, a wireless mini-endoscope that uses a 2 g wireless transmitter and a LiPo battery. Barbera et al. (2019) also introduced a wireless mini-endoscope that is powered by a battery backpack anchored on the animal’s back and that is equipped with Micro SD card storage. They were able to record the activity of medium spiny neurons (MSNs) in the dorsal striatum of two freely behaving and interacting mice. In both studies, power was supplied to the microscope by miniature LiPo batteries, which depending on their size, can provide power for 30 min to 1 h at the expense of increasing the weight of the microscope to several grams (Liberti et al., 2017). For future developments, local recording remains a very good solution for reducing the weight of the system. Transferring data samples at a very low frame rate (0.2–1 Hz) and adding a communication interface for the synchronization with external components such as behavior cameras would improve system feedback. The development of new wireless systems should also take into account future behavioral studies aiming to assess the impact of the weight and its distribution (e.g., backpack battery versus integrated battery) on animal behavior. Further improvements are required to optimize wireless mini-endoscopes, improvements that will largely benefit the neuroscience field by expanding the number of possible behavioral experiments that can be performed while monitoring brain activity. The potential of this technique can also be extended to animals that exhibit more complex social behavior like monkeys and small primates. In addition, more complex social behaviors can be assessed without the constraints of the cable from the imaging device.

## General Conclusion

Imaging neuronal activity using various sensors and actuators has emerged as a powerful tool that allows neuroscientists to record large-scale neuronal activity in freely behaving animals. Traditional tools used for imaging such as two-photon excitation microscopy have opened up the possibility of imaging Ca^2+^ activity patterns at the cellular and subcellular levels with unprecedented optical resolution. But these experimental setups are costly and require specific infrastructures that limit their dissemination among large numbers of laboratories. Also, these setups require the animals to be head-restrained during the imaging phase, which markedly limits the behavioral repertoire that can be tested. The development of mini-endoscopes has given rise to a new class of devices that have proven to be very useful in acquiring and correlating functional cellular signals from cortical and deep brain regions to animal behavior. The ease of use coupled with the portability and relative low cost of mini-endoscopes has provided a much-needed technical alternative. The capacity to take recordings from large neuronal populations in freely behaving animals has made it possible to analyze large-scale Ca^2+^ imaging data with higher statistical power and to discover different types of coding dynamics related to animal behavior. The versatility of these devices also provides a good platform for improvements to the original design based on research requirements. Designs that incorporate parallel electrophysiological recordings or that allow all-optical circuit-level investigations by combining imaging with optogenetic manipulations can be used to further dissect activity at the microcircuit level and increase our understanding of neural processes underlying animal behavior. The ability to construct lightweight small mini-endoscopes will contribute to animal well-being, will improve the read-out of neural activity under natural conditions, and will expand research to other animal models. By decreasing the size of these devices, mini-endoscopes now also permit the use of multiple devices on the same experimental subject, which allows for the imaging of multiple brain areas in mice and other larger animals such as rats and primates. This multi-site recording approach will open up new avenues for systems neuroscience research, where longitudinal large-scale recordings beyond a single local circuit can be performed and may uncover exciting new information related to circuit activity patterns governing brain-wide functional circuitry and synchronicity. Further improvements to these devices aiming to increase the depth of penetration in living tissue, fine tune spatial and temporal resolution, and add a wireless option for even less restrictive behavioral repertoires are needed to make functional imaging in freely behaving animals more widely accessible. The traditional use of mini-endoscopy is to image Ca^2+^ activity at the neuronal level. However, the advances in fluorescent reporters, onsite lens focus technologies, and two- and three-photon miniaturization will enable scientists to image cellular and subcellular compartments such as dendrites and dendritic spines. Further development of GEVIs, which have much more temporal resolution than GECIs, will push the use of mini-endoscopes even further, giving scientists the means to optically assess voltage changes across membranes and dendritic compartments. This will most likely revolutionize neuroscience research, giving scientists a tool to record, stimulate, and differentiate between the fast electrophysiological events associated with synaptic activity and the slow Ca^2+^ events associated with the various biomolecular processes that occur during learning and memory. In addition, mini-endoscopic imaging approaches may allow scientists to monitor activity related to spine formation/elimination, the dynamics of glia processes, microglia migration, and adult neurogenesis in freely behaving animals. The flexible methodological approach provided by mini-endoscopes will continue to afford neuroscientists with better tools to image large neuronal populations during increasingly less restrained behavioral paradigms. This, in turn, will lead to new datasets that will more accurately depict the relationship between recorded functional and structural activities and animal behavior.

## Author Contributions

All authors listed have made a substantial, direct and intellectual contribution to the work, and approved it for publication.

## Conflict of Interest

SD and HD declare a conflict of interest. SD is the owner of Doric Lenses and HD is an R&D scientist at Doric Lenses. The remaining authors declare that the research was conducted in the absence of any commercial or financial relationships that could be construed as a potential conflict of interest.
